# On Syphilis

**Published:** 1843-12-09

**Authors:** Meredith Clymer


CLINIC OF THE UNIVERSITY OF PENNSYLVANIA AT THE

### PHILADELPHIA HOSPITAL.

BY MEREDITH CLYMER, M. D.

(Reported for the Medical Examiner.)

### LECTURE I.

### ON SYPHILIS.

*Nov.* 29*th*, 1843.—Gentlemen:—The name Venereal Disease is given to all those morbid phenomena which result from impure sexual intercourse. Now, an immense mass of disorders is included in this comprehensive definition; but two great classes may be established, which occur occasionally combined, but which are always distinct, both in their nature and consequences; these are, I. Virulent Venereal Affections, recognising as an invariable proximate cause the direct action of a specific morbid poison. Chancre is the first manifestation of this class. II. Non-Virulent Venereal Affections, which do not necessarily result from the action of any one fixed cause. The prototype of this class is Gonorrhœa.

I shall to-day consider the Virulent Affections, or Syphilis. I shall not detain you by any historical disquisition to prove that syphilis has existed in all ages, or occupy your time by rebutting the evidence which has been adduced to bestow the honours of paternity on America. After an examination of the various theories on the origin of syphilis, you would probably acknowledge with Voltaire, that "*la verole est comme les beaux arts, on ne sait pas qui la inventée.*" But as I shall have frequent occasion to employ the term venereal or syphilitic virus, it is proper for me to inform you, by that expression I understand a morbid principle or poison, which applied under certain conditions to any portion of the human body, will determine there definite and characteristic phenomena; that this principle being absorbed and carried into the system, will, during the existence of the local or primary symptoms, and for an indefinite period subsequent to their cessation, contaminate the economy; and, finally, that this principle is capable of being transmitted hereditarily, and that, too, at a period when its presence in the system is not revealed by any external sign. This capability of hybernation for a number of years within the economy, without producing in the meanwhile any appreciable effect upon it, is a character peculiar to the syphilitic virus. The existence of a special virus was denied by Broussais and his School, who believed that chancres were nothing more than simple sores, and treated them accordingly. The existence of a specific morbid poison in syphilis can be demonstrated alone by its effects. Now the matter of a primary syphilitic sore or chancre introduced beneath the epidermis, is constant in its results, whoever the individual, or whatever the part of the body inoculated. This is conclusively proved by the numerous experiments of Ricord, Wallace, Mayo, Carmichael, Mairion, Parker, &c. No other secretion or product of an ulcerous or suppurating surface, will produce the same effects. I cannot, from want of time, enter at length into the vexed question of the identity or non-identity of the venereal poison; that is, whether the two classes of affections we have referred to are due to one virus, or result from the action of several. In my judgment, facts go to prove conclusively the non-identity of the virus of chancre and gonorrhœa, and to establish the validity of the distinction I have made. Gonorrhœal matter applied to a mucous surface will produce gonorrhœa, but in no instance is it capable of producing true chancre. It may act as an irritant, but it cannot produce a specific ulcer. The phenomena of constitutional syphilis never follow gonorrhœa. I know that the reverse has been frequently stated, and that, too, by very respectable authorities, but I will indicate to you the sources of error. In the cases which have been cited to prove the identity of the two virusses, it has been said that a diseased individual having had connection with several healthy persons, these latter would be variously affected; some would have chancres, some gonorrhœa, and others both together. Now in these cases, the two diseases coexisted in the diseased person. Had he or she been properly examined, larvated chancres would have been discovered either in the urethra, or about the neck of the uterus, or in the cul de sac of the vagina, or elsewhere. Never pronounce that a woman has no chancre until you have thoroughly examined her with a speculum. All those recorded cases, where no speculum was used, are perfectly worthless.

The classification of venereal diseases proposed by Ricord is as follows: I. Primitive or Direct, when they occur at the inoculated spot, from the immediate action of the virus. II. Successive, when they originate in the latter, and are produced elsewhere by absorption or contiguity of tissue, or accidental contact, as chancrous bubo, and the conversion of neighbouring abrasions, or leech bites into chancres. III. Secondary, when the skin and mucous membranes are affected after the reception of the chancrous matter into the system; and IV. Tertiary, when the cellular, fibrous, and bony structures are the seat of the constitutional symptoms. V. Diseases unconnected with syphilis.

This classification strikes me as unphilosophical, wanting in simplicity, and many of the grounds on which it is founded are incorrect and untenable. The two first may certainly, without violence, be included under one head; the second and third divisions are not susceptible of separation on the grounds given by Dr. Ricord. The so-called tertiary symptoms may arise without the necessary intervention of the secondary. Dr. Ricord asserts, that whilst the former may be transmitted hereditarily, the latter cannot be, except in a degenerated form, as scrofula. This assertion I agree with a large majority of the writers on syphilis, in denying, believing them to be both equally liable to propagation by inheritance. The fourth species, or non-syphilitic, I do not comprehend. A more simple and less objectionable classification is, I think, the following: I. Primitive or Local Syphilis, of which chancre is the only exponent. II. Consecutive, General, or Constitutional Syphilis, which is always strictly the consequence of a chancre.

By a chancre I mean a solution of continuity in the soft parts, produced by the disorganizing action of the syphilitic virus upon the inoculated spot. Chancre, wherever its seat, is the consequence of the application of a specific matter which a chancre alone secretes, and which produces a chancre whenever placed in circumstances favourable to contagion. Does any time elapse between the application of the virus and its action? Ricord is the only writer on syphilis who denies that there is a period of incubation, and on the ground that, in no instance after his experiments with inoculation did he observe any. When the virus is introduced beneath the epidermis its action may be immediate, but this is far from being the case when the infection is natural. The following case, mentioned by Dr. Huguier, one of the physicians of the Female Venereal Hospital at Paris, is one of many others which conclusively establish the possibility of incubation. Two friends after a debauch had connection with the same woman. Three days subsequently one was attacked with a chancre; and it was not until the twenty-first day that his friend became similarly affected, although he had in the meanwhile daily examined the parts, and suspended all sexual relations. The period of incubation varies with the degree of susceptibility of the organ, and the manner in which the affection was contracted.

The development of chancres may occur in three ways, 1. Pustule; 2, Ulceration; 3. Small abscesses. The physical characters of chancres vary with their number, and their location. If several chancres exist at the same spot the form is irregular, and the edges are jagged and notched; in the natural folds of the skin and mucous membranes, and in depending parts where the virus acts in a longitudinal direction, their form is of the same character. The tissue which is the seat of the chancre alters its aspect. On the lips, which are highly organised the chancre is granular; on the labia majora, and other cellular parts, the chancre is of a greyish appearance; when situated in the body of the penis, if the ulceration has penetrated to the fibrous envelope of the corposa cavernosa, the ulcer will present that white, shining, brilliant appearance peculiar to fibrous tissue. When on the glans penis the granulations are generally of a bright red, and bleed on the slightest touch. The edges of a chancre are generally perpendicular, and look as if they had been cut out by a sharp instrument, the primitive venereal virus nearly always acting centripetally. Such is the appearance of the sores in the man I now present to you, who has a number of small chancres in his prepuce, combined with a balanitis and a posthitis. If, however, the virus comes in contact with previous abrasions, the cellular tissue is more rapidly destroyed than the skin or mucous membrane, and the latter are dissected up, as in the case now on the table. The character of the pus varies with the period that the ulcerations have existed, at first serous, thin, even sanious, and mixed with the disorganising tissue; then healthier, thicker and often mixed with blood; and it finally becomes consistent, of a yellowish tinge, of a better character, resembling that on the surface of a blister in full suppuration. It varies too with the nature of the ulcer. There are two stages in chancre, one of ulceration and the other of cicatrisation. Now there is one important difference in these two stages, which you should notice, as it is of great practical utility. In the first stage a specific matter is secreted, which retains its inoculable properties. The specific period has no absolute limits of duration. Dr. Ricord has seen it in one instance last for seven years, and inoculate at the end of that time. The usual duration, is from one to two months. In the second stage the chancre ceases to be inoculable, granulates and heals. If a chancre is in the second stage, in the progress of cicatrisation, connection will not be followed by disease; but if the ulcer is in the first stage, inoculation will inevitably result. Chancres may be divided according as their location is external or concealed; hence we have *external* and *larvated* chancres. Larvated, or concealed chancres in the male are situated in the urethra, and in the mucous fold of the prepuce, and at the base of the glans where there is phymosis; in the female, in the urethra, at the orifice of the vagina, above the carunculæ, in the vagina, and in and about the neck of the uterus; and within the anus, in the rectum, in the fauces, larynx &c. in both sexes. Chancres may also be divided according to their characters. I admit four varities. 1. Follicular; 2. Iudurated; 3. Phagadenic; 4. Furunculous.

*A Follicular chancre* is enveloped either in a sebaceous or hair follicle, or in the tonsillary structure of the vagina. On the application of the virus, the follicle becomes inflamed, more salient, and its point elevated; it is filled with an opaline fluid, which finally becomes pus. If exposed to the air, a scab forms, if not, the summit becomes flattened and cupped, and the ulceration extends from half a line to a line in depth. Several little ulcers generally exist at the same time adjoining each other, they unite, and their edges become irregular and jagged. When the ulcer is a hair follicle, it is usually deeper, and the hair which for some time retains its place, finally falls out. In the third variety of follicular chancre, the virus acts immediately on the glandular structure of the vagina, and according to Huguier there is no pustules, but ulceration at the commencement.

The indurated chancre was first described by John de Vigo. John Hunter described it as the type of the primary venereal ulcer, and from him it has been called the Hunterian chancre. Ricord asserts that a chancre never becomes indurated before the fifth day, I am quite sure that a chancre is never indurated ab initio, but that it may become so at any period of its progress. Ricord, considers induration as the index of the constitution being affected, and I am inclined to believe him correct. It is certainly the most annoying of all forms of chancre, and the most sure to be followed by constitutional symptoms. This fact you should bear in mind; it is of immense importance therapeutically. The induration is due to the effusion of plastic lymph into the cellular tissue, or as Ricord believes, into the lymphatic capillaries. The late Dr. Fricke of Hamburg, proved conclusively, that the application of corrosive sublimate to the prepuce will occasion ulcers with a hard base. Now, the only safe means of diagnosis between these and true chancre is their non-inoculable character, and their never being followed by constitutional symptoms. The indurated chancre is usually very indolent. Induration begins at the base of the chancre. Benjamin Bell compared it to a split pea, inserted in the tissue, so cartilaginous is the hardness. It is usually round when situated in homogeneous tissues with regular edges, and is of a very dark colour. The base may be indurated and not the edges, and the reverse, but usually the whole chancre is equally compromised. Immediately around it ulcers may exist, which do not present any of these characters. You will usually meet with this form of chancre on the edge of the prepuce, on the corona glandis, on the edge of the labia majora, and especially on the labia minora. On the lips and tongue, the indurated chancre may be confounded with cancer, and vice versa. An indurated chancre rarely or never disappears spontaneously.

*A Phaghadenic chancre* is usually very rapid and destructive in its progress, increasing in extent and not in depth, and accompanied with severe pain. Its march is irregular and serpiginous. It occurs generally in constitutions worn out by intemperance, and follows very often irritating dressings which have been injudiciously applied to irritated or inflamed chancres, especially mercurial ointment. It is called the black slough in England.

There is a form of phagadenic sore, called diptheritic or pultaceous, which is exceedingly chronic, (Ricord has seen it last for seven years,) and is covered either entirely, or partially by a pultaceous diptheritic secretion. The base is œdematous, and the edges are elevated, irregular, and serrated, and it is surrounded by a dull purple areola, and it increases by successive ulceration of the depending parts. The constitution becomes seriously implicated, and the patient finally sinks. This form of chancre occurs in ill-fed, badly lodged individuals, in whom there is previous organic disease.

*The Furunculous chancre*, is the consequence of the subcutaneous or sub-mucous abscesses, previously described. Chancerous buboes, may also be included in this category. This form of chancre generally burrows in the cellular tissue, destroying it to some extent.

Chancres may be modified by inflammation, the occurrence of erysipelas, and by gangrene. This last is a very serious complication, the destruction of the part inevitable, and deformity always follows cicatrisation. Ricord says, that gangrene neutralises the virus, and constitutional symptoms rarely occur.

The seat of chancre is next to be considered. Its ordinary seat in the two sexes is the genital organs, and the other parts of the genital apparatus most commonly affected are, in men, the penus the base of the glans, the base of the mucous fold of the prepuce as it touches the collun, the edge of the prepuce, the glans, the meatus, the fossa navicularis, the urethra, the body of the penis, the scrotum and the pubes. In the female, the orifice of the vagina at the inferior part, especially the fourchette, and the sides and depressions of the carunculæ, the nymphæ, the labia, the meatus, the clitoris and the pubis. The nymphæ are especially liable to be the seat of chancre both from their organisation and situation. They are covered throughout with mucous membrane, which absorbs with great readiness, and they contain a vast number of sebaceous follicles, which are ready recipients for the venereal virus. The part they perform in coition will naturally suggest to you the great risk of contamination they are exposed to. After the genital organs, the most common seat of chancre is the perineum and the pourtour of the anus. There is no part of the body to which the virus can gain direct admission but may be the seat of chancre. The progress of civilization, and the depraved and irregular taste which too often accompanies it, render all parts of the body liable to chancre. I would willingly pass over this part of my subject, for I feel pained in exposing the gross depravity of humanity, but the interests of science are paramount to all other considerations and require that I should indicate to you the localities where you may occasionally meet with chancre. I am happy to state that the occurrence of chancre in these illicit spots is very rare in this country. I have frequently seen primary syphilitic ulcers in the French hospitals, in the anus, lower extremity of the rectum, on the lips, the tongue, chin, tonsils, velum palati, between the breasts, in the axilla, the nipple, &c. I am unwilling to enter more in detail, but bear in mind this fact, that there is no part of the body which may not become the victim of a disordered taste; not even the toes and fingers. Swediaur relates the case of a man who, having passed the night between two women, one at the head and the other at the foot of the bed, to revenge himself for the inactivity he was condemned to, touched with his foot the organs of one of the women; the consequence was a chancre on his great toe, followed by indurated inguinal ganglion. The manner of propagation of a chancre can only be in one way—by means of the virus. Every other imaginable method has been devised, from the promiscuous use of holy water, public bathing, &c., to a tight boot. But sexual intercourse, although the most common, is not the only mode of contagion. During labour a mother may inoculate her child; the surgeon's finger, if abraded, may become inoculated by examining a chancre. A melancholy instance of this kind occurred a short time ago in Paris. Dr. Hourmann, a physician of celebrity, attached to the Female Venereal Hospital, inoculated a cut on his finger, whilst examining a patient; consecutive symptoms supervened, and he died at the end of ten months. Surgical instruments employed in opening chancres, buboes, &c., may also communicate the disease; but these are all examples of inoculation; the difference is in the vehicle.

The diagnosis of chancre is sometimes very difficult. Most respectable and honourable persons, who would scorn an untruth under all other circumstances, will frequently deceive you in this respect without any hesitation, and deny stoutly the possibility of the fact. Women are especially positive, and often *impayable*. A sense of shame will induce many to resort to untruth to conceal their errors. Locality is, as you have seen, no argument against the existence of chancre. Indurated chancre may be confounded with cancer, and vice versa, especially in the tongue. The employment of a syphilitic treatment is, in many cases, the only means of diagnosis. The sources of error are numerous; for all sores on the genitals are not syphilitic, but merely simple abrasions or wounds. A recent suspicious connection will, of course, throw much light on the subject.

The prognosis is rarely grave when the constitution is tolerably good, and the sore is not phagadenic. An indurated chancre may be regarded as a certain sign of the constitution being infected.

Before proceeding to the local treatment of chancre, I want to give you the description of a chancre induced by artificial inoculation, which the experiments of Ricord have made so famous. During the first twenty-four hours after the introduction of the virus beneath the epidermis, there is slight inflammation, followed on the second day by tumefaction, and a small ecthymatous papule, surrounded by an areola; from the third to the fourth day there is a vesicle, filled by a fluid more or less opaque, with a dark point at the apex; about the fourth or fifth day this becomes purulent, with the centre depressed, and resembles a small-pox pustule. The areola, which up to this period had increased, now diminishes, and after the fifth day the surrounding tissues, hitherto unaffected, become of a cartilaginous hardness. After the sixth day the pustule dries, becomes covered by a laminated crust, and this, when detached, becomes a characteristic ulcer with a broad base.

*Prophylaxis.*—I have not here time to enter into an examination of the various prophylactic measures which have been proposed. The most certain one is the avoidance of the cause. Cleanliness is another means of prevention. The presence of the catamenia and vagino-uterine catarrh is said to diminish the risk of infection in women. Urination, and careful larvation of the organ, is a prophylactic measure in the man.

*Treatment.*—A chancre at first is a purely local affection, and the virus is not yet carried into the system, and the constitution is not yet compromised. Now the greater the length of time the specific sore is allowed to remain, the greater are the chances of constitutional contamination. Your first object should, therefore, be to convert the specific ulcer into a simple one, by destroying the seat of the virus, unless there is some positive contraindicating symptoms present. This is best accomplished by some caustic application. If the sore be a recent one, if induration has not occurred, you will probably prevent subsequent infection of the system. Now various escharotics have been proposed to accomplish this. The solid nitrate of silver is the one commonly used by Ricord, and preferred by him and most surgeons. You usually pass the crayon of nitrate of silver, previously cut to a sharp point, over the edges and fundus of the sore, cauterising it freely. Now, in some persons great irritation and inflammation will follow the use of the lunar caustic. This I have often seen happen. It occurred in the case of the man with the chancrous bubo that I exhibited to you. Before the interne left the ward, great tumefaction and inflammation had supervened, so as to alarm him very much. The nitrate of silver is by many considered as powerful enough to irritate, but not to destroy. The object of the escharotic employed is to disorganise the whole of the contaminated tissue. For this purpose there is, perhaps, no better caustic than the proto-nitrate of mercury, made by dissolving a drachm of the sub-nitrate of mercury in an ounce of nitric acid; a camel's hair pencil, or a piece of lint attached to a handle, is saturated with the caustic, and the ulcer is freely painted over; a yellow eschar results, which, in separating, leaves a simple ulcer beneath.

Strong nitric acid, and the acid nitrate of silver, (made by dissolving an ounce of the nitrate of silver in an ounce of nitric acid,) have also been recommended. I have no experience with either of these escharotics in the treatment of chancre. The potassa cum calce has been strongly recommended by Mr. Langston Parker, of Birmingham, a surgeon of much experience in Venereal Diseases, and the author of an excellent treatise on them. He forms it into a paste of moderate consistency with the spirits of wine just before using it, and covers the sac with it. He allows it to remain on from half a minute to a minute, according to the degree of pain. It is then removed by means of a bone spatula, and the black eschar left treated in the usual manner presently to be indicated. The aggregate amount of pain he states is less than that of the lunar caustic, and the effect much more certain. When the sore is quite recent and small, I have frequently succeeded in effecting a rapid cure by the application of a pencil of the sulphate of copper, until the surface of the sore is destroyed. I then direct the patient to use frequent ablutions of cold water during the day to the part. The eschar will be detached in the course of a few days, with diminution in the size of the ulcer, which generally heals rapidly without any troublesome symptoms. Cauterisation must be renewed until the ulcer begins to granulate.

After cauterisation rest must be generally enjoined, and a poultice is to be applied; if there be much inflammation lead water, &c., must be used, together with low diet, &c. When the eschar separates, the sore must be treated as a simple ulcer, by weak stimulating lotions, as solutions of the sulphate of copper, the black and yellow washes; the sulphate of alumina, &c. Never use ointments or other greasy applications, especially mercurial ointment, to a chancre. One of the best applications to a chancre in this stage is the aromatic wine of the French Pharmacopœia. This consists of a number of the aromatic herbs digested in port wine. The wine used here is double the strength of the French preparation, owing to port being used instead of Burgundy. It should, therefore, be always diluted one-half at least. Tannin may be added to render it more astringent. If the chancres are very irritable and painful, use an aqueous solution of opium, belladonna, and other anodyne lotions; a solution of the acetate of lead powerfully controls pain in these cases.

Larvated urethral chancres may be cauterised by means of Llallemand's instrument, and afterwards treated by the introduction of a piece of lint into the canal, and injections. Sometimes cauterisation is decidedly contraindicated, by the presence of great irritation, or actual inflammation, or the chancre may be complicated with phymosis, and you may not be enabled to reach it. Now, in the former of these cases you must employ strict antiphlogistic treatment. Rest and proper diet are always powerful adjuvants in the treatment of syphilis. The former cannot always be followed, but the latter always. If inflammation exists, and you use caustics or stimulating dresses, sloughing may ensue, and small ulcers will assume from the same cause a phagadenic character, and irreparable mischief may ensue. The anti-mercurial treatment of syphilis has proved the great beneficial results arising from rest, proper regimen, and soothing applications in controlling the ravages of the disease. In both the instances mentioned you must first treat the local complications, and subsequently, if necessary, resort to the specific treatment for the constitutional effect. This plan of treatment will occupy our attention at our next meeting.

